# *Ab initio* study of alloying of MnBi to enhance the energy product

**DOI:** 10.1039/d1ra05007a

**Published:** 2021-09-17

**Authors:** Tula R. Paudel, Bhubnesh Lama, Parashu Kharel

**Affiliations:** Department of Physics, South Dakota School of Mines and Technology Rapid City SD 57701 USA tula.paudel@sdsmt.edu; Department of Physics, South Dakota State University Brookings SD 57707 USA

## Abstract

High energy density magnets are preferred over induction magnets for many applications, including electric motors used in flying rovers, electric vehicles, and wind turbines. However, several issues related to cost and supply with state-of-the-art rare-earth-based magnets necessitate development of high-flux magnets containing low-cost earth-abundant materials. Here, by using first-principles density functional theory, we demonstrate the possibility of tuning magnetization and magnetocrystalline anisotropy of one of the candidate materials, MnBi, by alloying it with foreign elements. By using density functional theory in the high-throughput fashion, we consider the possibility of various metal and non-metal elements in the periodic table occupying empty sites of MnBi and found that MnBi-based alloys with Rh, Pd, Li, and O are stable against decomposition to constituent elements and have larger magnetization energy product compared to MnBi. Combined with other favorable properties of MnBi, such as high Curie temperature and earth abundancy of constituent elements, we envision the possibility of MnBi-based high-energy-density magnets.

## Abstract 2

The DFT calculations show that filing empty interstitial sites of MnBi leads to stable alloys with Li, O, F, Sc, Y, Rh, Pd among which alloy with Li, O, Rh, Pd and Pt have larger magnetization and enhanced energy products (up to 20%) than bulk MnBi.

## Graphical abstract

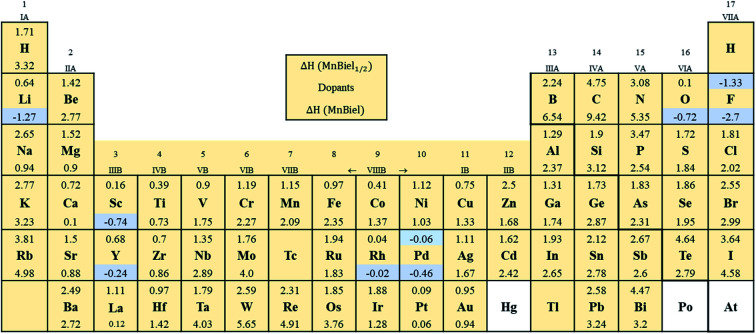

MnBi, a member of Mn–A alloys, where A can be any element with 3+ oxidation state, such as Al, Ga, In, has potential to be a good magnet.^1–4^ The compound has sizeable magnetocrystalline anisotropy energy (∼0.163 meV uc^−1^ (ref. 5)), coercivity (∼7 kOe at room temperature^6^), and energy product as large as 17 MGOe,^5^ which are very attractive for magnet applications. Additionally, the compound has a large Curie temperature of 628 K,^1^ and both coercivity and magnetocrystalline anisotropy increase with increasing temperature making it well suited for high-temperature applications. However, even with such favorable properties, the MnBi and MnBi-based compounds suffer from relatively low saturation magnetization.^7^ Various ideas, including defect engineering,^2,8–13^ exchange coupling with soft magnets,^7,10,14–21^ and microstructural engineering,^22–26^ have been tested to improve the energy products in these compounds. However, they have so far yielded mixed results. While most of the doping with various foreign elements and microstructure refinement has shown an increase of coercivity at the expense of magnetization,^2,3,8,27^ Sn doping^3^ is found to increase magnetization and magnetic anisotropy, leading to an increase in energy product.

In this manuscript, we test an alternative approach of alloying MnBi with foreign elements to enhance magnetization and magnetocrystalline anisotropy, leading to enhanced energy product (*BH*)_max_. We use the high throughput density functional theory for screening suitable elements to form an alloy with MnBi. The structure of MnBi offers clues on why alloying may be possible in MnBi. The low-temperature phase of the MnBi crystallizes in hexagonal NiAs type structure (space group no. 194), as shown in Fig. 1. The lattice vectors of such a crystal are 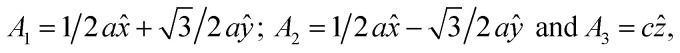 where *a* and *c* are lattice constants. Mn occupies 2*a* Wyckoff's positions with *D*_3d_ site symmetry: 0 and (1/2*A*_3_); Bi occupies 2*c* Wyckoff's positions with *D*_3h_ symmetry (1/3*A*_1_ + 2/3*A*_2_ + 1/4*A*_3_) and (2/3*A*_1_ + 1/3*A*_2_ + 3/4*A*_3_) while the other high symmetry Wyckoff's 2d positions: (2/3*A*_1_ + 1/3*A*_2_ + 1/4*A*_3_) and (1/3*A*_1_ + 2/3*A*_2_ + 3/4*A*_3_) are empty. We incorporate foreign elements to these sites and search for the elements that increase magnetization and coercivity. We consider two cases for each elemental (el) insertion. (i) Only one of two vacant sites is filled with foreign elements (half-filled case) such that effective concentration of alloy is 0.2/uc; the other half-filled configuration obtained by filling the rest vacant position is expected to behave similarly because of the similarity of lattice symmetry and the local environment surrounding the elements when placed in those positions, and (ii) both of the vacant sites are filled (full-filled case) such that effective concentration of alloy is 0.33/uc. Our strategy to find the suitable element for alloying is first to evaluate the formation energy of each alloy to find the stable one; second, determine saturation magnetization of stable alloys; and finally, calculate magnetic anisotropy energy of alloys that are stable and have magnetization larger than undoped MnBi.

**Fig. 1:**
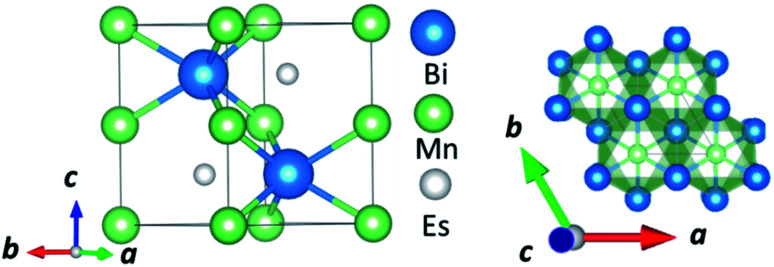
Side view (left) and top view (right) of the NiAs type crystal structure of low-temperature phase MnBi.

For the stable alloy, the formation energy, Δ*H*, must be negative, which ascertains energy gain by alloying. The formation energy of the MnBi:el is evaluated as Δ*H* = *E*(MnBi:el) − *E*(MnBi) − *E*(el). Here *E*(MnBi) and *E*(el) are energies of MnBi and energy of element, el. Similarly, *E*(MnBi:el) is the energy of the configuration of the alloy. We determine the energy of the alloy by comparing the energies of two configurations: first, ferromagnetic (FM) configuration in which magnetic moment of alloying elements aligns parallel to the magnetic moments of Mn; and second antiferromagnetic (AFM) configurations in which the magnetic moment of alloying element is aligned anti-parallel to the magnetic moments of Mn. The calculated formation energy from these calculations is likely to be in the order of ∼0.1 eV per formula unit, corresponding to ∼10 kJ mol^−1^.^28^

Fig. 2 shows the formation energy of the alloy with the various elements in the periodic table. Out of all those calculations, the stable alloys are the ones with the negative formation energy. In the half-filled case, only F and Pd doped alloys have negative formation energies. These are marked with light color in Fig. 2. Similarly, in the full-filled case, F, Y, and Rh doped alloys also have negative formation energies, indicating the alloys to be stable with respect to decomposition to the elemental phase. Other half-filled alloys with Rh, Pt, O, and Sc have formation energies less than 0.2 eV; these dopants only partially occupy these vacant sites. We calculate site occupancy *N*_occ_/*N* = exp(−Δ*H*/*k*_B_*T*) at 500 K, where *N* is the number of available sites, *k*_B_ is the Boltzmann constant, and *T* is the temperature. We found that Rh, Pt, O, and Sc occupy 40%, 25%, 24%, and 2% of empty sites, respectively. Similarly, full-filled MnBi alloys with La, Ca, and Pt have slightly positive formation energy (<0.2 eV) and only partially occupy the empty sites: La occupies 6%, Ca occupies 9%, and Pt occupies 23% of the empty sites. When Δ*H* > 0.2 eV occupation of interstitial sites of MnBi reduces to less than 1%. While ternary phase diagrams of many of these elements with MnBi are unavailable, experimental studies including Mn–Rh–Bi ternary phase diagram shows the presence of various phases including Mn_1.05_Rh_0.02_Bi,^29^ Mn_0.8_RhBi_0.2_,^30^ and Mn_5_Rh_6_Bi_18_ and MnRhBi_3_ (ref. 31) consistent with our prediction of high solubility of Rh in MnBi.

**Fig. 2:**
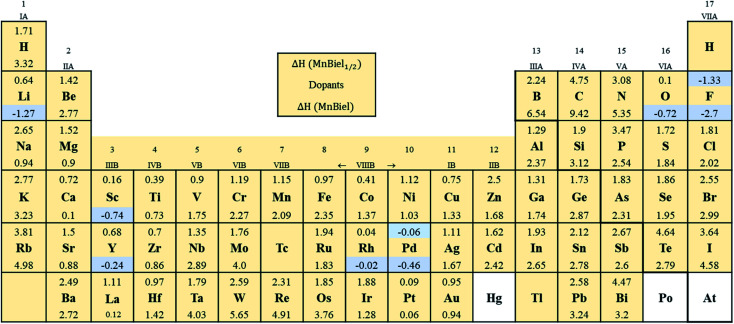
The formation energy (eV) of the MnBi alloy with the various elements. The numbers above and below each element in the box represent the formation energy, Δ*H*, of half-filled (MnBi:el_1/2_) and full filled (MnBi:el) alloy with the element (el). The isolated box shows the legend. The highlighted elements form a stable alloy with MnBi.

All the alloys with negative formation energy are found to have a larger volume compared to that of MnBi. The lattice volume increases, but modestly, according to the increase in the atomic radius.^32^ The smallest volume increase is observed in F (1%), and the largest volume increase is observed in the case of Y (42%). Volume increases more in the full-filled than half-filled case; however, the increase is not linear; for example, the volume increase of full-filled MnBiRh is ∼19%, whereas that of half-filled MnBiRh_1/2_ is ∼14%. This nonlinear increase in volume indicates additional hybridization between the alloying elements and MnBi while both empty sites are occupied. Despite the increase in volume, the volume formation, which is defined in a similar way as the formation energy, Δ*V*_F_ = *V*(MnBi:*n*el) − (*V*_MnBi_ − *nV*_el_), where *n* is number of doped elements, Ve_l_ is volume of doped element, and *V*_MnBi_ is the volume of MnBi, is generally negative (except for MnBi:Pt) as shown in Table 1. The negative Δ*V*_F_ also indicates bonding of the alloying elements with Mn and Bi in MnBi.

**Table 1:** Change in saturation magnetization (Δ*M*), anisotropy (ΔAE), volume formation (Δ*V*_F_), *c*/*a*, energy difference (Δ*E* = *E*_FM_ − *E*_AFM_) between antiferromagnetic (AFM) and ferromagnetic (FM) configurations, estimated Curie temperature (*T*_c_) and change in energy product, Δ(*BH*)_max_ (%) as a function of alloying elements (el) in half-filled MnBi:el_1/2_ and full-filled MnBi:el alloys

Case	Δ*M* (%)	ΔAE (MJ m^−3^)	Δ*V* (%)	Δ*V*_F_ (Å^3^)	*c*/*a*	Δ*E* (eV)	*T*_c_ (K)	Δ(*BH*)_max_ (%)
MnBi	0	0			1.33	−0.3	580	0
MnBi:Li	9	4	18	−23.26	1.24	−0.1	190	18
MnBI:O	9	5	8	−19.62	1.05	−0.29	555	18
MnBi:F_1/2_	−2		1.0	−18.23	1.28			
MnBi:F	−7		1.5	−19.62	1.27			
MnBi:Sc_1/2_	−9		26	−0.10	1.18			
MnBi:Sc	−2		36	−16.15	1.18			
MnBi:Y	−7		42	−26.15	1.17			
MnBi:Rh_1/2_	8	3.0	13	−1.53	1.33	−0.27	522	16
MnBi:Rh	14	0.92	19	−10.80	1.21	−0.16	312	32
MnBi:Pd_1/2_	9	20.7	18	−0.46	1.33	−0.24	457	18
MnBi:Pd	14	0.33	27	−5.98	1.22	−0.17	327	30
MnBi:Pt_1/2_	10	6.37	19	2.74	1.25	−0.23	442	21
MnBi:Pt	10	4.79	20	−13.04	1.20	−0.22	417	21

Having found the stable alloys, we next analyze their magnetic properties. Table 1 shows changes in magnetization with respect to the 3.54*μ*_B_ per Mn of bulk MnBi. Here magnetization per Mn atom is determined by dividing total magnetization density that minimize total energy of system by number of Mn atoms, assuming magnetism is mostly carried by Mn atoms. Out of the alloys with negative formation energy, alloying with F, Sc, Y reduces the net magnetization, where alloying with Li, O, Rh, Pd, and Pt increases net magnetization by up to 14% compared to the undoped case. In the case of F doping, F-p orbital hybridizes strongly with Bi-p orbitals (Fig. 3c), which couples to Mn-d orbitals antiferromagnetically like that in bulk MnBi, thereby reducing the net moment. In the case of Sc and Y, their 3d-states, as shown in Fig. 3d and e, mainly lie in the conduction band; thus, they hybridize with Mn minority d-states, inducing small moments in themselves and reducing moments in Mn, and the moments in Y and Sc couple antiferromagnetically with Mn moments further reducing the overall magnetizations.

**Fig. 3:**
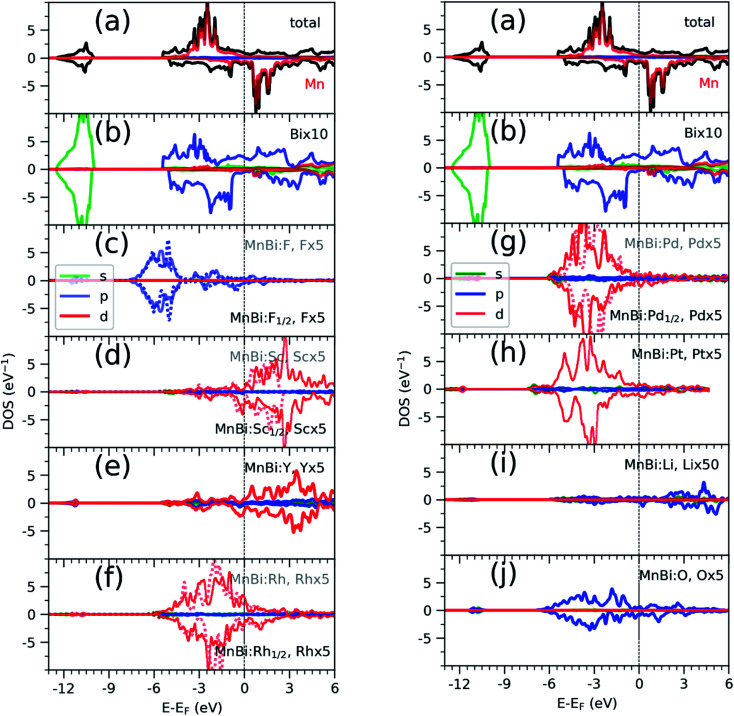
Density of states (DOS) of MnBi total (black) projected to Mn atoms (a), projected to Bi atoms (b), and projected to various alloying elements (el) (c) to (j) resolved according to spin: majority in positive axis and minority in negative axis and orbitals: color red, blue, and green corresponds to d, p and s orbitals respectively. In the cases (F, Rh and Pd), where both half-filled and full-filled alloys are stable, the DOS of element in full-filled alloy is shown with dotted line in the background of solid line that corresponds to DOS of the element in half-filled case. el × *x*, where *x* is 5 or 10 in (c–f) and (g–j) corresponds to the enlargement factor for DOS for better visualization.

Alloying with Rh, Pd, Pt increases the net magnetization of the system. 4d orbitals of the Rh (Fig. 3f) and Pd (Fig. 3g) and 5d orbitals of the Pt (Fig. 3h) lies mainly in the valence band and hybridize strongly with the majority spin-channel of Mn, which induces small magnetic moments on themselves and enhances magnetic moments on Mn. These moments on Rh, Pd, and Pt, however, couple ferromagnetically with Mn moments, which is opposite to interaction between Mn at the regular sites and interstitial sites, which couples antiferromagnetically,^33,34^ or interaction between Sc, Y in interstitial sites and Mn at regular sites, as we discussed previously. The difference comes from the large exchange splitting of Mn-d bands leading to almost no interaction between electrons with different spins located at the different sites. However, in Rh, Pd, and Pt, even though intra-atomic exchange interaction leads to d-orbitals splitting according to spin like that in Mn, the splitting is much smaller. As a result, bands corresponding to both spin channels are occupied though there is a slight shift between them, which results in a small magnetic moment. Additionally, they have a significant overlap with Mn d-orbitals occupied by the majority spin channel, resulting in ferromagnetic direct exchange.

Alloying group II elements like Li and O also increases overall magnetization. The s-states of Li and p-states of O lie on the opposite side of the Fermi level: The O-p states lie primarily in the valence band as shown in Fig. 3j, while Li-s states lie in the conduction band as shown in Fig. 3i. As a result, the hybridization between Li-s states and Mn-d states, and O-p and Mn-d states is spin-dependent; the oxygen states hybridize mostly with the Mn-d majority states, while Li-s states hybridize mostly with Mn-d minority states leading to the enhancement of Mn moments and development of small moments in Li and O themselves. The magnetic moments of Li and O also couple ferromagnetically because of direct exchange with Mn moments and help increasing overall magnetization. We verify this hypothesis by explicitly comparing the energy of the system with the moments of Mn and moments of Li and O coupling ferromagnetically and antiferromagnetically and found that the ferromagnetically coupled system has lower energy. Using the energy difference Δ*E* = *E*_FM_ − *E*_AFM_ we estimate the Curie temperature in the mean-field approximations by using *T*_c_ = Δ*E*/3.^34^ For the bulk MnBi, we calculate Δ*E* = 150 meV per Mn and *T*_c_ ≈ 580 K, which is reasonably close to extrapolated experimental value of 775 K;^35^ the structural transition at 628 K hinder measuring real *T*_c_ of MnBi. Using the same approach, we evaluate the *T*_c_ of stable MnBi alloys with enhanced magnetizations. The second to last column of the Table 1 shows the calculated results, which shows that *T*_c_ reduces in general upon doping. This is consistent with the fact that upon doping anti-ferromagnetic magnetic exchange *J*_1_ between nearest neighbor Mn-atoms in *c*-directions^34^ increases because of enhancement of volume, reduction of *c*/*a* ratio and increase in orbital overlap. The other exchange parameters related with second (*J*_2_), third (*J*_3_), fourth (*J*_4_), fifth (*J*_5_) and sixth (*J*_6_) nearest neighbor Mn atoms are ferromagnetic and smaller than *J*_1_.^5^ With the increase of *J*_1_ the net exchange *J* = 2*J*_1_ + 6*J*_2_ + 12*J*_3_ + 2*J*_4_ + 2*J*_5_ + 2*J*_6_ can be expected to decrease and along with it the *T*_c_, as 
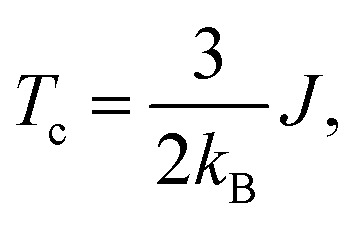
^5^ while the other *J*_s_ are also expected to be affected upon alloying, their large coefficients will remain same so that *J* remains negative and alloy still is ferromagnetic.

Next, we calculate change in magnetic anisotropy energy MAE, ΔMAE = MAE(MnBi:el/el_1/2_) − MAE(MnBi), in stable alloys with magnetization larger than that of bulk MnBi. MAE is computed by taking the energy difference between the system with magnetization pointing along [100] direction and the system with magnetization pointing along [001] direction, MAE = *E*(*M*‖[100]) − *E*(*M*‖[001]). The results in Table 1 show that anisotropy energy increases upon alloying compared to MnBi (∼1 MJ m^−3^) in all cases. Since the anisotropy energy *E* = *K*_1_ sin ^2^*θ* + *K*_2_ sin ^4^*θ*, where *θ* is the angle between easy axis [001] and the direction of magnetization, and *K*_2_ ≪ *K*_1_, ΔMAE roughly corresponds to change in *K*_1_. We note that magnetization of MnBi alloy with Rd and Pd_1/2_ points along [001] direction, similar to the bulk MnBi, the magnetization of MnBi alloyed with Li, O, Pd, and Pt changes to in-plane [100] direction. Qualitatively, such changes in MAE and easy axis are related to subtle changes in band distribution near the Fermi level and may require separate detailed investigation. The magnetic anisotropy is a relativistic effect driven by spin–orbit coupling (SOC). As the spin–orbit coupling energy is much smaller than the bandwidth of Mn-3d states, perturbative expansion of energy can be used to explain the spin–orbit coupling effect. The lowest non-zero correction to the energy in such expansion includes the term proportional to 1/(*E*_unocc_ − *E*_occ_) where *E*_occ(unocc)_ is the energy of occupied (unoccupied) state around Fermi energy for each *k*-points^36^ in the Brillouin zone. While the system is metallic, *E*_unocc_ − *E*_occ_ remains finite for the majority of the *k*-points and the SOC induced change in energy does not diverge. The non-zero DOS of alloying elements near the Fermi level indicates the possibility of such changes near Fermi levels.

Finally, we estimate the theoretical value of maximum energy product (*BH*)_max_ assuming applied field *H* much smaller than magnetization, *M*. In this approximation, magnetic induction field *B* = 4π*M* in CGS units, and remanent magnetization, *M*_r_ = *M*_s_, saturation magnetization when the magnetic hysteresis loop is almost rectangular. The (*BH*)_max_ = *B*_r_^2^/4, where the remnant field *B*_r_ = 4π*M*_r_ = 4π*M*_s_.^37^ The formula allows us to estimate the energy-product using only the intrinsic properties such as actual magnetization (emu cm^−3^) and homogeneous sample without using coercivity that relies on the extrinsic factors such as shape and size of the sample. The last column of Table 1 shows the calculated percentage change in energy product to calculated energy product of 20 MGOe, which is similar to the value of 17 MGOe^38,39^ measured experimentally. The energy product enhances when MnBi is alloyed with Pd, Pt, Rh, Li, and O per the change in magnetizations.

In summary, we used the first-principles density functional theory to find an alloy with the increased magnetization and anisotropy. We predict MnBi alloy with Pd, Pt, Rh, Li, and O are stable against decomposition to constituent elements and have larger magnetization and anisotropy compared to MnBi and have a high energy product. Magnetic easy axis of MnBi alloy with Pd_1/2_ and Rh remains the same as bulk MnBi and lies out-of-plane direction, while the magnetic-easy-axis of MnBi alloy with Li, O, Pd and Pt lie in-plane. Pd can rotate the magnetic-easy-axis^40^ from in-plane to out-of-plane depending upon the percentage of Pd that would be incorporated in MnBi. We anticipate this comprehensive study of MnBi alloy spurs more theoretical and experimental study. We note that the alloying of these elements with MnBi may require non-equilibrium growth methods as their formation energies are relatively small.

## Computational methods

We employ the projected augmented wave (PAW)^41^ method for the electron-ion potential and the gradient density approximation (GGA) for exchange–correlation potential, as implemented in the Vienna *ab initio* simulation package (VASP)^42,43^ with the recommended set of pseudopotentials.^44^ The calculations are carried out using the kinetic energy cutoff of 340 eV and 6 × 6 × 1 *k*-point mesh for Brillouin zone integration of pseudocubic unit cells. The *k*-points are scaled according to size. We fully relax ionic coordinates with the force convergence limit of 0.001 eV per atom. For the anisotropy calculations, we include an additional onsite Coulomb interaction Hubbard (*U*–*J*) parameter of 3 eV to Mn-3d states so that the sign of magnetocrystalline of bulk MnBi is consistent with the experiments and includes spin–orbit coupling terms explicitly.

## Data availability

The data that support the findings of this are available from the corresponding author upon reasonable request.

## Conflicts of interest

There are no conflicts to declare.

## Supplementary Material
